# Polyvinyl alcohol/soy protein isolate nanofibrous patch for wound-healing applications

**DOI:** 10.1007/s40204-019-00120-4

**Published:** 2019-09-24

**Authors:** Bahareh Khabbaz, Atefeh Solouk, Hamid Mirzadeh

**Affiliations:** 1 Biomedical Engineering Department, Faculty of Engineering, Islamic Azad University Central Tehran Branch, Tehran, 13185/867 Iran; 2 Biomedical Engineering Department, Amirkabir University of Technology (Tehran Polytechnic), Tehran, 15875-4413 Iran; 3 Polymer Engineering and Color Technology Department, Amirkabir University of Technology (Tehran Polytechnic), Tehran, 15875-4413 Iran

**Keywords:** Soy protein isolate, Electrospun nanofibrous mat, Wound healing

## Abstract

Soy protein isolate (SPI), due to its biocompatibility, biodegradability, abundance and being inexpensive, is a suitable polymer for medical applications. In this study, electrospun nanofibrous mats (ENMs) and casting films (CFs), comprising polyvinyl alcohol (PVA)/SPI, were prepared and compared. Both crosslinked ENMs and CFs physical, chemical, mechanical, and biological properties were investigated for wound-healing applications. Considering the importance of exudate absorption by wound dressing the uptake test of all samples was performed in simulated exudate solution. The amount of absorbed exudate, water vapor transmission rate, and mechanical elongation for CFs were 69.243% ± 22.7, 266.7 g/m^2^ day, and 2.0825% and increased to 383.33% ± 105.3, 1332.02 g/m^2^ day, and 12.292% in the case of ENMs, respectively. There was no significant difference between cell supporting of the two samples due to similar composition and their non-toxic properties. The results showed that ENMs have promising potential in wound-healing applications.

## Introduction

In recent years, there is a growing interest in using naturally derived biomaterials to reduce the use of petrochemical feedstock and manufacture high-quality, cost-effective, biodegradable, and biocompatible products. From this aspect, these materials from renewable resources will be an eco-friendly substitute for petroleum-based materials (Koshy et al. 2015). Merits including bioactivity, biodegradation, and presence of natural binding sites for manipulating the cell adhesion and growth both in vitro and in vivo have made animal- and plant-based proteins ideal biomaterials (Chien et al. 2013). Soybean is widely available in the world and is considered as a cost competitive feed stock polymer. There are three forms of commercial soy protein based on the extraction process and each has different protein content; defatted soy flour, soy protein concentrate, and soy protein isolate (SPI) containing approximately 53%, 74%, and 93% protein, respectively. All these soy products are representatives of biodegradable ‘‘green’’ composites due to their good performance (Cho et al. 2010).

Through the production of oil, the obtained product from defatted soy flakes is SPI which is the purest form of the protein and has a minimum of 90% protein content. Due to its abundance, biodegradability, biocompatibility, and being inexpensive, SPI is a suitable polymer for medical applications (Shankar et al. 2013). Amino acids of aspartic acid (aspargine) and glutamic acid (glutamine), non-polar amino acids (glycine, alanine, valine, and leucine), basic amino acids (lysine and arginine), and less than 1% of cysteine are presented in the chemical structure of the soy protein also make an advantage for biomedical application of SPI. Soy protein can be modified through chemical, physical, and enzymatic modifications for a variety of biomedical applications due to the presence of high amounts of reactive groups, such as –NH_2_, –OH, and –SH. Having a greater storage stability in comparison with other biodegradable polymers and natural proteins, as well as being of non-animal origin (plant derived), are additional advantages of soy protein for biomedical applications (Ramji and Shah 2014). SPI-based products are formed through denaturation of the protein structure from native state and reformation of new configurations through new linkages within the protein molecule. Changes in pH, electrical force, mechanical force, or heat are the possible ways that denaturation can be induced (Koshy et al. 2015).

Polyvinyl alcohol (PVA) is a synthetic polymer that has features such as being semi-crystalline, water soluble, biodegradable, and biocompatible with human tissues. This polymer is produced in the largest quantity worldwide (Taghizadeh and Sabouri 2013). Contact lenses, drug delivery devices, wound dressings, orthopedic devices, and artificial organs are some examples of PVA used in various pharmaceutical and biomedical applications. It can be crosslinked by chemical agents such as glutaraldehyde (GA), acetaldehyde, and formaldehyde (Ye et al. 2014). To fabricate biodegradable composites, PVA can be combined with natural polymers. This blended PVA/natural polymer has a couple of advantages SUCH AS biodegradability, biocompatibility, chemical resistance, and excellent physical properties which makes it a promising biomaterial in many fields of industrial applications (Su et al. 2010a, b).

An ideal wound dressing should possess some characteristics such as non-toxicity, biocompatibility, easy application, appropriate water vapor permeability, and optimal water uptake. A minimum adherence to the wound bed which provides an easy removal of the wound dress, so it would not cause a secondary trauma to the regenerated skin. Furthermore, a suitable microenvironment is necessary that a wound dressing should provide for wound healing (Saeed et al. 2017; Doulabi et al. 2015). Depending on the injury location and the type of the exudates, the mechanical and physiochemical properties of the wound dressing should suit the condition with adjustments (Moghadas et al. 2016). In this aspect, blend polymers are more beneficial in providing more previously mentioned requirements in comparison with a single polymer, since their properties are improved (Doulabi et al. 2015).

Recently, electrospinning with capability to form polymeric fibers, of different diameters, has gained attention as fabrication technique for preparation of fibrous biomaterial. The morphological resemblance to native extracellular matrix (ECM), high surface/volume ratio, high porosity, and pore interconnectivity is amongst the main features of electrospun structures (Khorshidi et al. 2016a, b).

Previous studies showed that the compatibility of the PVA/SPI blend could be enhanced by the presence of glycerol and/or greater modification of the interfacial adhesion (Su et al. 2010a, b). However, these blends are unstable in contact with aqueous media. To prevent the dissolution of these blends in aqueous media and considerable loss of mechanical strength, crosslinking procedures are necessary (Khorshidi et al. 2016a, b).

The aim of this study is the fabrication and evaluation of films and nanofibers based on polyvinyl alcohol/soy protein isolate using solvent-casting and electrospinning methods for the purpose of wound healing. Different studies of blending these two polymers have been carried out either in the form of electrospinning (Cho et al. 2010; Zhang et al. 2003) or film formation (Su et al. 2008), but to the best of our knowledge, no work in the literature has been reported the composition of PVA/SPI/glycerol for the purpose of wound healing.

## Materials and methods

### Materials

PVA (*M*_w_ 72000 g mol^−1^, the degree of hydrolysis ≥ 98%) was purchased from Merck. Food grade SPI powder (batch no: E24) containing ≥ 90% protein and ≤ 7.0% moisture was purchased from FOODCHEM. Analytical grade glycerol (*M*_w_ 92.09) with ≥ 99% purity was purchased from MP Biomedicals. Laboratory grade nonionic surfactant Triton X-100 (t-Oct-C6H4-(OCH2CH2)*x*OH *x* = 9–10) was purchased from Sigma. Analytical grade sodium hydroxide (NaOH) pellets were used to prepare a 1 M solution at room temperature. Dialdehyde-crosslinking agent of glutaraldehyde (25 wt% in H_2_O) was purchased from Merck. Laboratory grade acetone with ˃ 99% purity and hydrochloric acid with ≥ 37% purity was purchased from Ghatran ShimiT.Co.IRAN.

### Film preparation

Films of PVA/SPI/glycerol were formed using the solvent-casting method. PVA aqueous solution (10 wt%) was prepared by dissolving PVA in deionized water in a water bath at 70 °C for 2 h. SPI aqueous solution (8.5 wt%) was prepared by stirring in deionized water at room temperature for 10–15 min. To form SPI-based films, the protein structure needs to be denatured. Here, the denaturation of SPI induced by altering the pH by adding 1 M NaOH to the solution and applying heat. Therefore, SPI solution was stir-heated in a water bath at 80 °C for 30 min with the adjusted pH (7.88 ± 0.12). These two solutions were mixed with a mass ratio of 50:50, and to acquire a uniform solution, it was stirred in a water bath at 70 °C for 1 h. The amount of 5 wt% of the plasticizer (glycerol) was added into the final solution. To obtain the equal thickness, 5 mL of solutions were poured into 8 mm diameter polystyrene petri dishes and cooled to room temperature to remove bubbles. Finally, aqueous resins were oven dried at 50 °C for 6 h (Su et al. 2010a, b).

### Electrospinning

Solution preparation for the process of electrospinning was carried out precisely the same as the preparation of the solution for CFs with the same amount of polymers. To have a spinnable solution, denaturation of the native state of the protein has to occur, and thus, the disulfide, hydrogen, ionic bonds, and steric and hydrophobic interactions have to be broken down (Zhang et al. 2003). For this purpose, again, 1 M NaOH was added into the SPI solution and heat was applied. PVA and SPI solutions were mixed with a mass ratio of 50:50, so that a homogeneous solution was prepared. Triton X-100 at a ratio of 5 wt% on the basis of PVA mass was added into the final solution to reduce the surface tension of water and delay the gelation of PVA, since water as a solvent would lead to the formation of beads in the process of electrospinning. Again, 5 wt% of the glycerol on the basis of the final solution was added.

A 5 mL plastic syringe was loaded with the final solution to put in the electrospinning machine (Fanavaran Nano-Meghyas(. An 18-gauge needle was used and connected to the positive charge of the power supply. The voltage of 25 kV was applied and the distance between the tip of the needle and the collector was set to 15 cm. Speed of the rotatory collector was chosen to be 300 rpm. The solution was infused by amicropump toward the collector at a rate of 0.5 mL/h. This process was carried out at room temperature (Cho et al. 2010).

### Crosslinking

Since these constructs are made of hydrophilic-based materials, they need to be prevented from dissolution upon hydration. ENMs dissolved immediately after immersion in water, whereas it took time for CFs to be dissolved. Yet, CFs were susceptible to dissolution in water. Hence, GA was used as a cross-linker agent for immersion of the specimens in its solution.

In immersion method, 5% GA was diluted by acetone with the proportion of 10:90 (v/v) (10 refers to 5% GA and 90 refers to acetone). Since GA activates in an acidic environment, a sufficient amount of HCl was added to the solution to adjust the pH. CFs and ENMs were immersed in the solution of 5% GA/acetone (10:90 v/v) for 12 h and then were put in an oven at 50 °C for another 12 h. After that, to remove the unreacted GA, samples were washed in distilled water for 2 h on a shaker. Every hour, the distilled water was renewed with a fresh one (Qiu 2012).

### Scanning electron microscopy (SEM)

To observe the morphology and microstructure of the CFs and ENMs, and cell adhesion, SEM (Seron Technologies Inc AIS2100) with the accelerating voltage of 15 kV was used.

### Fourier transform infrared (FTIR) spectra analysis

FTIR spectra were obtained for SPI powder, PVA powder, CFs, and ENMs with and without glycerol, uncrosslinked and crosslinked PVA/SPI/glycerol CFs and ENMs. FTIR measurements were recorded in the range of 4000–400 cm^−1^ at a resolution of 4 cm^−1^ using 40 scans on the Nicolet Nexus spectrometer.

### Water contact angle (WCA)

Water contact angle in the air on the surface of crosslinked CFs and ENMs was measured based on the ASTM D724-99 standard. The WCA was evaluated as the average value of measurements made on the opposite side of water drops (*n* = 5).

### Simulated exudate solution uptake

Fluid absorption by wound dressing is an important factor in their functionality in chronic wound treatment. Solution A is known as a standard solution for evaluation of fluid uptake by the wound dressing that has a viscosity equivalent to water (Lovett et al. 2006). Hence, simulated thin (Solution A) wound exudate was prepared to evaluate the solution uptake of the CFs and ENMs. Chemicals used for modeling thin wound exudate solution are presented in Table 1. After measuring the initial weight of the specimens (*m*_d_), they were put in contact with Solution A. At specific times, the superficial moisture of the samples was removed using a filter paper, and then, they were weighed (*m*_w_). The measurements were carried out for 48 h and the water absorption of the samples (*n* = 3) was calculated based on the following equation:

1 $$ {\text{Simulated exudate solution uptake}} \,\left ( \% \right) = \frac{{m_{\text{w}} - m_{\text{d}} }}{{m_{\text{d}} }} \times 100         . $$

**Table 1:** Composition of the simulated wound exudate solutions

Simulated thin exudate (solution A)
1000 g water
0.368 g calcium chloride
8.298 g sodium chloride

### Water vapor transmission rate (WVTR)

The permeability of the CFs and ENMs toward moisture was evaluated by the water vapor transmission rate measurements across the specimens. The penicillin vials with the inner diameter of 1.3 cm were filled with 8 mL PBS (*n* = 3). The crosslinked CFs and ENMs were cut into a circular shape to cover the exposure area of the vials and were fixed onto them using a parafilm. The initial weight of the assemblies was measured prior their placement in an incubator at 37 °C. A graph of water evaporation versus time was plotted by measuring the weight of the assemblies every hour. An open cup vial was considered as the control to simulate a condition in which no dressing would be applied. Measurements were carried out until at least six points gave a straight line (*R*^2^ ≥ 0.99). The WVTR values were evaluated using the following formula: (water loss rate signifies the slope):

2 $$ {\text{WVTR}} = \frac{{{\text{slope}} \times 24}}{\text{area}}\left( {\frac{g}{{m^{2} {\text{day}}}}} \right). $$

### Mechanical strength

Specimens were prepared for tensile test. Samples were cut into a dog-bone shape of 1 × 6 cm for CFs and 1 × 3 cm for ENMs (*n* = 3). The sample’s thickness was measured prior its test using a micrometer with a sensitivity of 1 µm. The test was performed at room temperature using a cross-head speed of 5 mm/min for CFs and 1 mm/min for ENMs. The percentage of elongation-at-breakpoint and ultimate tensile strength (UTS) were determined by Instron 5566 tensile tester.

### Cell responses

An ideal material must be non-toxic and biocompatible even its degraded products. Hence, the CFs and ENMs were evaluated to study the effect of these structures on living cells by the tests, as described below.

#### Cell adhesion

L929 mouse fibroblast cells were thawed and cultured in DMEM (Dulbecco’s Modified Eagle’s Medium) supplemented with 10% fetal bovine serum and 1% (v/v) penicillin–streptomycin. Cells were cultured in a humidified atmosphere of 5% CO_2_ at 37 °C in an incubator. When the cells reached the confluence of 70%, they were detached using trypsin enzyme and seeded into the 12-well plates.

To evaluate cell adhesion and morphology, crosslinked CFs and ENMs were sterilized. Both types of samples were washed in 70% ethanol twice and several times in phosphate buffer each for 15 min. After that, samples were immersed in culture medium for 12 h. 300 µL of cell suspension with the intensity of 10^5^ cell/cm^2^ was added to the wells containing samples and medium (*n* = 3). They were kept in an incubator for 48 h; then, CFs and ENMs containing cells were prepared for observation of cell adhesion using SEM.

#### Cell viability

Indirect contact method was used for the evaluation of cell viability and the values were obtained using the formula mentioned at the end of this part. Primarily, crosslinked CFs and ENMs had to be sterilized. For this purpose, samples were washed in 70% ethanol twice and then immersed in PBS (phosphate buffer solution) for 15 min for 3 times. After sterilization, they were immersed in the culture medium at a ratio of 6 cm^2^/mL (*n* = 3). Plates containing both samples and culture medium were put in an incubator for a 24-h extraction. A suspension of cells with the intensity of 5 × 10^3^ was seeded into each well of a 96-well plate. After 72 h of cell culture in the presence of sample extracts, the cell viability was assessed using MTT (3-(4,5-dimethylthiazol-2-yl)-2,5-diphenyltetrazolium bromide) assay. The optical density (OD) of the samples and control (the control sample was a tissue culture polystyrene plate) were measured at 570 nm using a spectrophotometer:

3 $$ {\text{Cell viability}}\,\left ( \% \right) = \frac{\text{mean OD of samples}}{\text{mean OD of negative control}} \times 100. $$

### Statistical analysis

Excel software was used for statistical analysis. Comparison made between more than two groups was performed using ANOVA method. All errors and error bars present standard deviation (SD) from the mean. OriginPro version 9.3 was used to draw graphs. The contact angle and fiber diameter measurements of ENMs were carried out using Image J software and 100 fibers were chosen randomly to calculate the average fiber diameter.

## Results

**Fig. 1:**
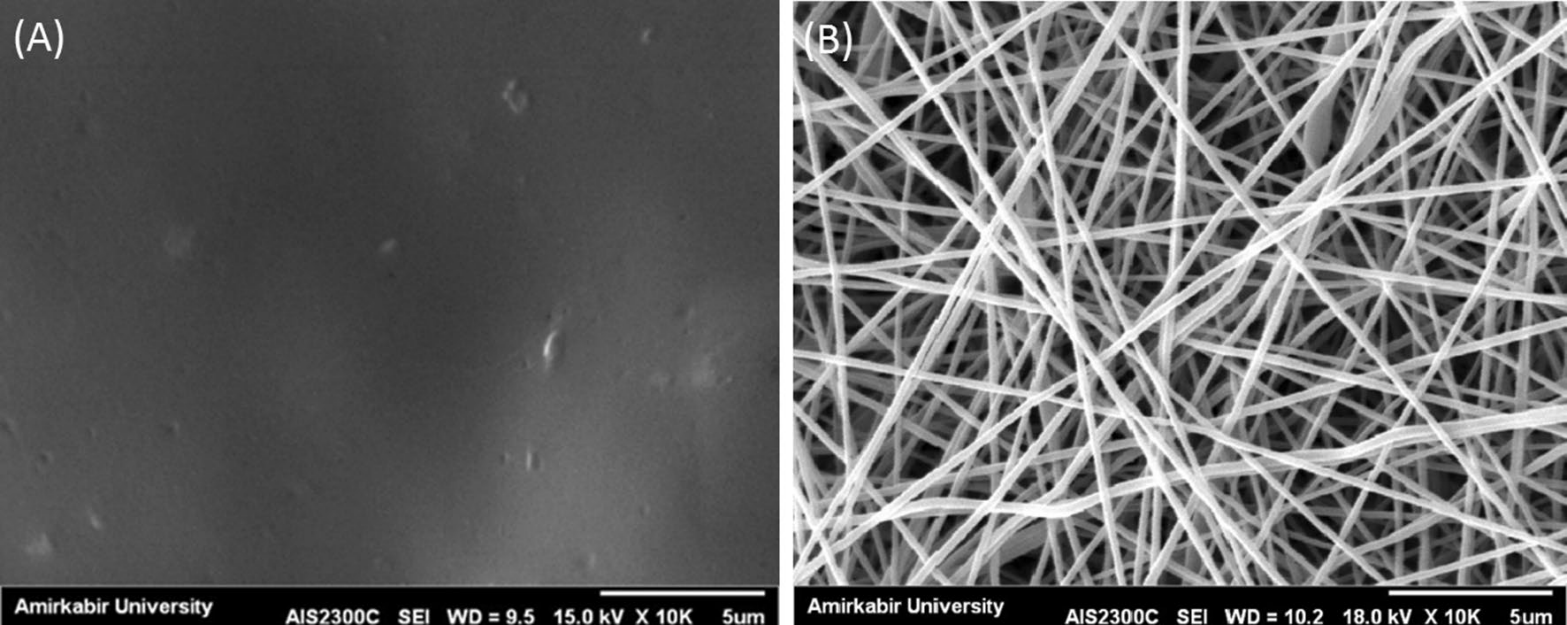
SEM images of **a** CF and **b** ENM

CFs were transparent, flexible and they were in light yellow. They possessed a smooth surface and were peeled off easily from petri dishes. The thickness of the CFs was 50 µm at most. SEM images of CFs showed a dense and almost uniform structure (Fig. 1a).

**Fig. 2:**
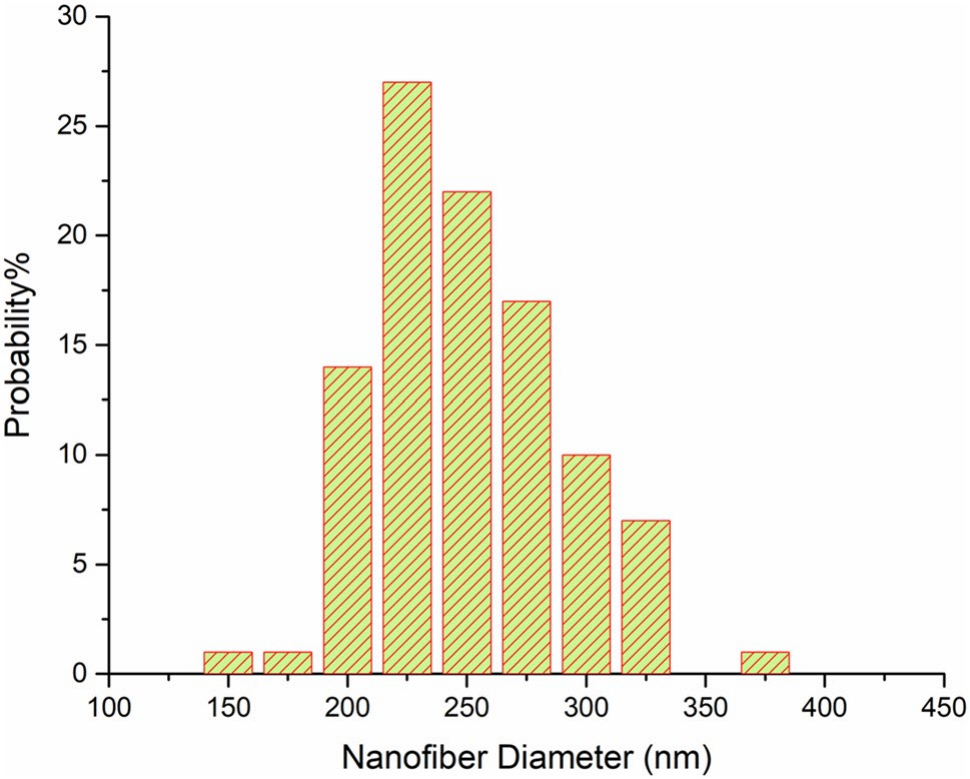
Fiber diameter distribution graph of ENM

SEM images showed that ENMs were bead free and the chosen concentrations of the materials were suitable to form electrospun fibers (Fig. 1b). The solution was not able to be electrospun without the presence of Triton X-100 and it would be sprayed. The electrospinning parameters including the distance between the tip of the needle and the collector, the applied voltage, and the flow rate were proper, as well. Figure 2 shows the distribution of fibers diameter. The average fiber diameter with the presence of glycerol was 238.69 $\pm$ 38.73 nm.

**Fig. 3:**
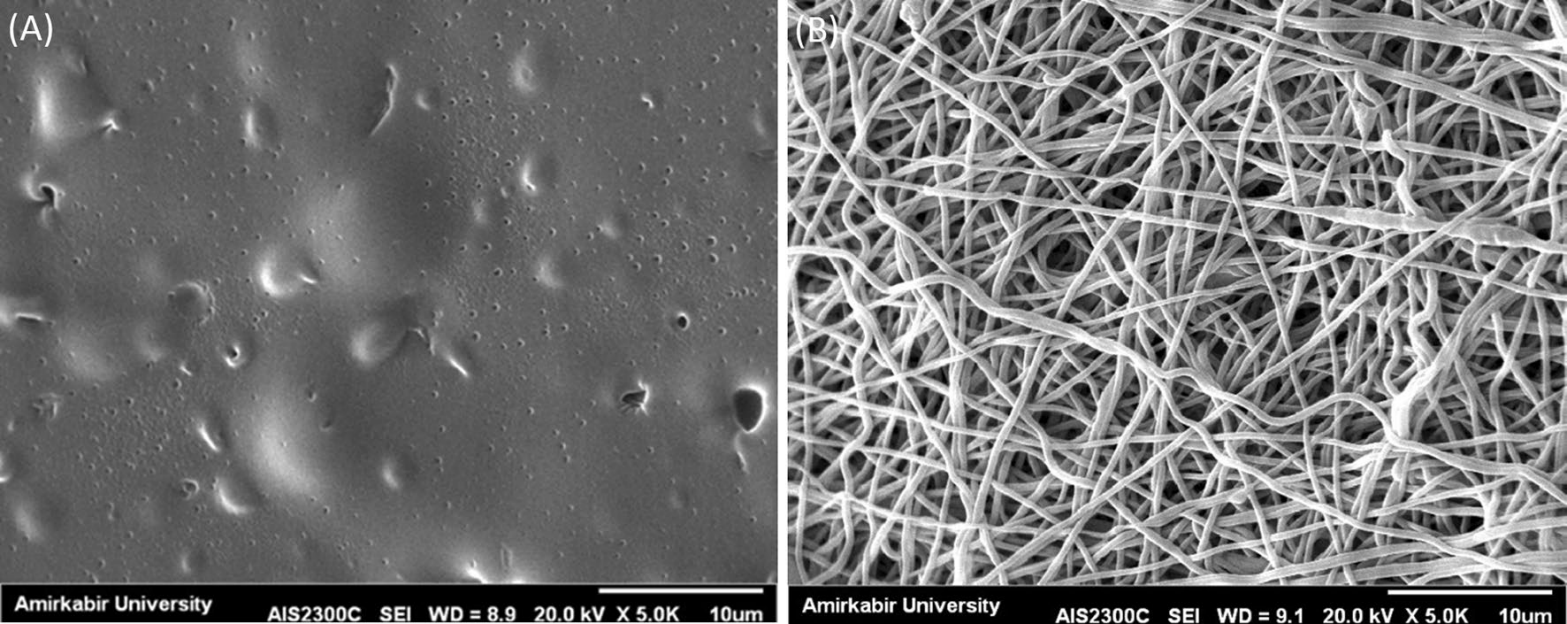
SEM images of **a** CF and **b** ENM after crosslinking in diluted GA/acetone solution

Immersion in diluted GA solution by acetone showed a satisfying result. Hence, changes in morphology were studied. SEM images showed fine pores in the morphology of CFs crosslinked by diluted GA/acetone solution. The average fiber diameter of ENMs after crosslinking resulted in thicker fibers (1.5 times) (Fig. 3a, b).

### Fourier transform infrared spectra (FTIR) analysis

**Fig. 4:**
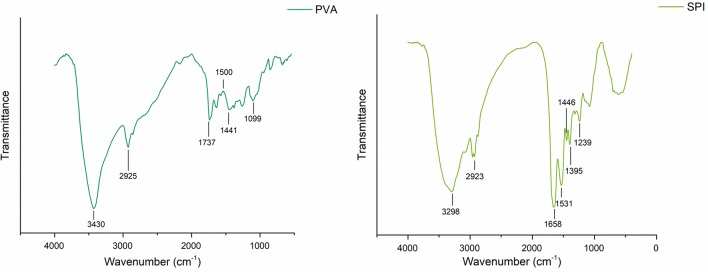
FTIR spectra of PVA and SPI powder

Figure 4 shows FTIR spectra of PVA and SPI powder, respectively. Peaks at the range of 3550–3200 cm^−1^ refer to the stretching O–H from intramolecular and intermolecular hydrogen bonding. Stretching C–H from alkyl group is shown in the range of 3000–2840 cm^−1^ and peaks around 1750–1735 refer to the C=O and C–O bands of polyvinyl alcohol’s acetate group. In addition, it was reported that in pure PVA, there is a peak at 1500 cm^−1^ which is caused by the deformation vibration of –CH_2_ in –CH_2_OH. The C–O–C band and CH_2_ are represented in the range of 1150–1085 cm^−1^ and 1461–1417 cm^−1^, respectively (Mansur et al. 2008).

In SPI powder, the amino acid group has a peak at 3300 cm^−1^ and stretching C–H band is vibrated at 2850 cm^−1^. By precisely looking at amid groups in SPI powder, the determined peaks at the range of 1700–1600 cm^−1^ refer to the stretching C=O of amid I band and peaks at the range of 1575–1480 cm^−1^ refer to the bending N–H of amid II. Peaks at the range of 1472–1241 attribute to the (C) O–O, stretching C–N band, bending N–H of amid III band, and the presence of β sheets in the structure of soy protein. The broadening peak at the range of 3500–3000 can be attributed to the free and connected N–H and O–H bands that enable the protein structure to form a hydrogen bond with the carbonyl group of peptide bond (Koshy et al. 2015; Ramji and Shah 2014; Su et al. 2010a, b).

**Fig. 5:**
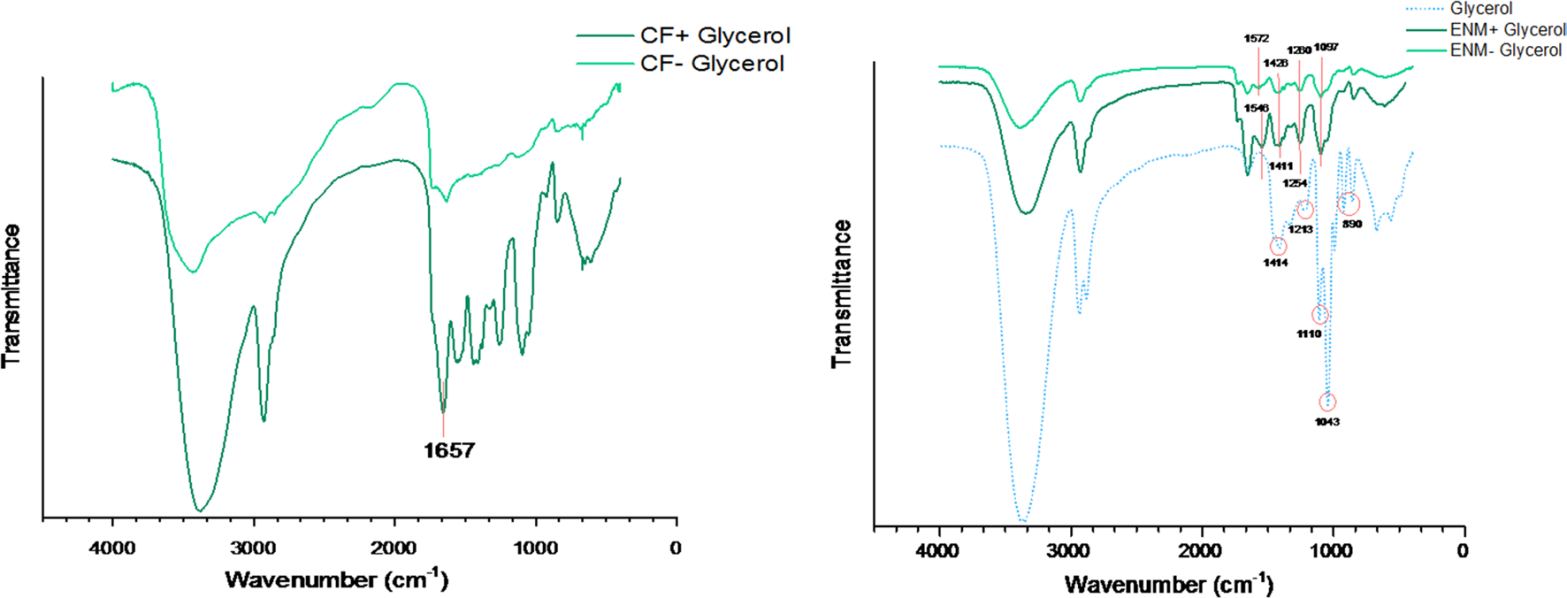
FTIR spectra of CF and ENM with and without glycerol

FTIR spectra of both CFs and ENMs with glycerol are shown in Fig. 5. When glycerol is not in the structure, the vibration at the range of 1600–1400 cm^−1^ and 1250–1150 cm^−1^ refer to the stretching N–H, stretching C–N, and bending N–H of amid III bands. There is a slight relocation of O–H band in the spectrum which can be attributed to a special chemical interaction occurring between PVA and SPI. With the presence of glycerol in the structure, the intensity of the peaks increased significantly and this increase was more dramatic in CFs spectrum. It was reported that glycerol has 5 known peaks in the fingerprint region from 1150 to 800 cm^−1^. Relative peaks of formation of the ester bond between glycerol and SPI is obvious at 1657 cm^−1^ (Su et al. 2010a, b).

**Fig. 6:**
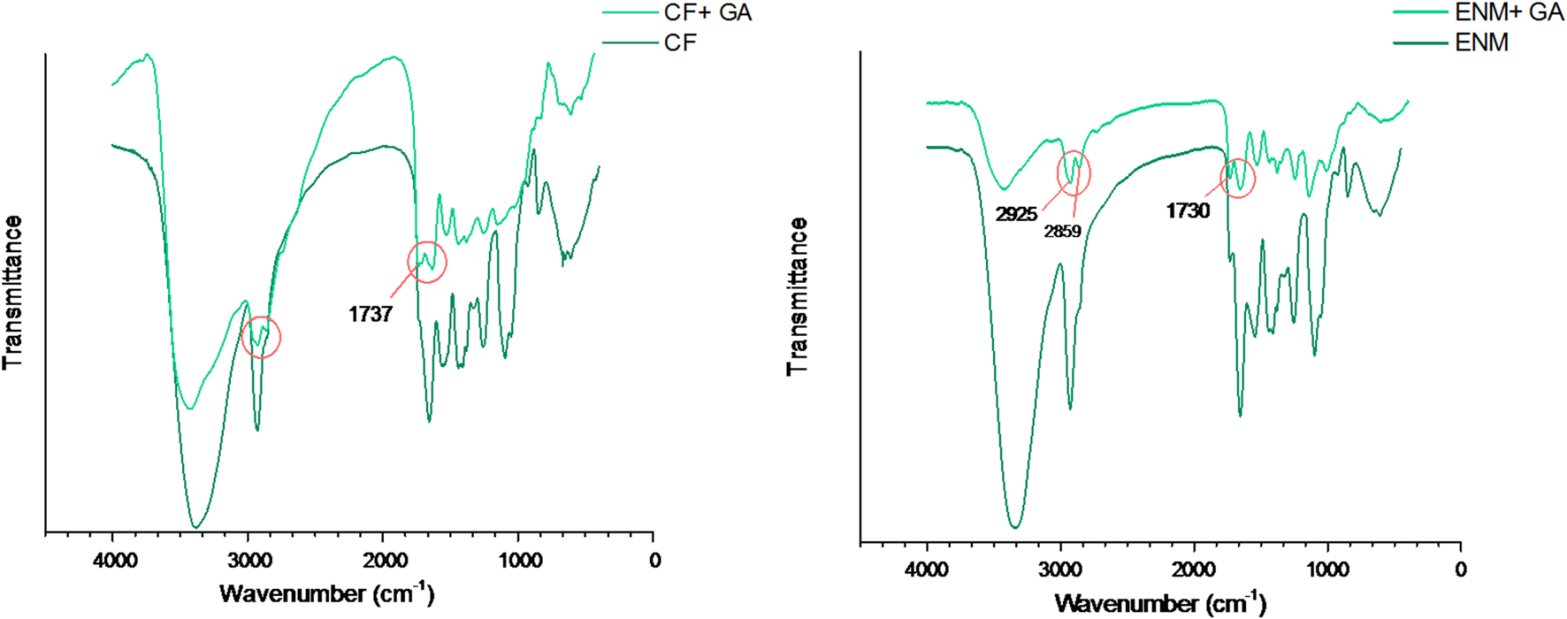
FTIR spectra of CF and ENM with added GA

FTIR spectrum of crosslinked specimens by immersion in GA/acetone solution is shown in Fig. 6. A decrease in the intensity of hydrogen bond at 3200–3650 cm^−1^ was observed and it can be attributed to the formation of acetal bridge in the polymeric chain. Two stretching vibrations of C–H from alkyl and O=C–H from the aldehyde were observed between 2730 and 2860 cm^−1^. In addition, the presence of GA was confirmed in the broad region of 1720–1740 cm^−1^ which refers to the carbonyl group (Mansur et al. 2008). This spectrum was recorded before washing the specimens in removing the unreacted GA.

### Water contact angle (WCA)

One of the biocompatibility criteria of a biomaterial is surface hydrophilicity. Surface hydrophilicity is an important indicator of a proper cell-surface interaction which can be appraised by water contact angle test (Bahrami et al. 2019). Contact angle measurement is a good indicator of the CFs and ENMs hydrophilicity. No angle was formed on the surface of the ENMs. This can be attributed to the porous structure of the nanofibers that the drop of water penetrates into the construct; therefore, measuring the WCA was not possible and no angle was formed. Hence, due to increased porosity, the permeability of the samples against water droplets has increased. All the values for CFs were in the range of 0 ˂ *θ* ˂ 90, so they can be considered as hydrophilic. The contact angle value of the CFs was 72.79˚ ± 4.7.

### Simulated exudate solution uptake

**Fig. 7:**
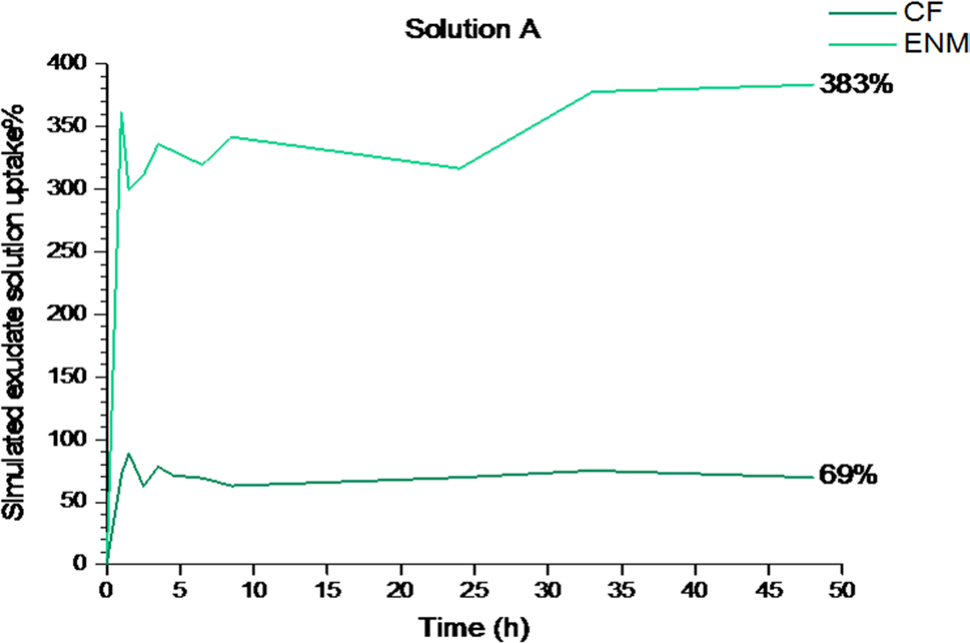
Simulated exudate solution uptake percentage of the CF and ENM in “solution A” after 48 h

Providing a moist environment for a wound bed is critical for the purpose of wound healing. This criterion can be determined by assessment of the fluid uptake and water vapor transmission rate through the patch. To provide gaseous exchange and absorption of wound exudate, some degree of fluid uptake is desirable (Peles and Zilberman 2012). The simulated exudate solution uptake percentage of the CFs and ENMs in “solution A” after 48 h was 69.243% ± 22.77708 and 383.33% ± 105.324, respectively. In other words, the water uptake of ENMs was 5.5 times more than water uptake of the CFs. The graph of simulated exudate solution uptake percentage versus time was plotted (Fig. 7). The simulated exudate solution uptake pattern of the ENMs was irregular. The constant simulated exudate solution uptake was achieved after 33 h.

### Water vapor transmission rate (WVTR)

**Fig. 8:**
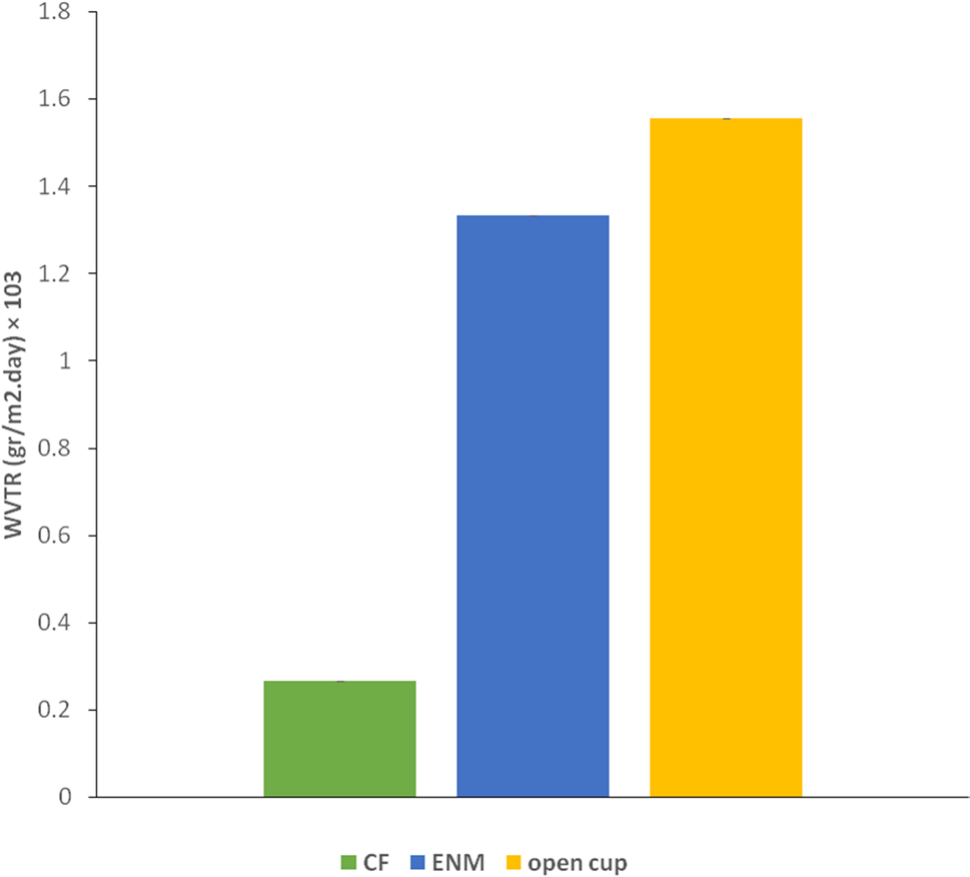
Water vapor transmission rate (WVTR) values of the CF, ENM, and open cup

A good management of WVTR is crucial as an effective wound patch, as it will provide a moist environment for the course of wound healing. Wound dehydration may occur as a result of excessive WVTR, whereas the bacterial infection and maceration may be the result of low WVTR (Peles and Zilberman 2012). The water loss caused by evaporation through the CFs and ENMs is linearly dependent on time (*R*^2^ ≥ 0.99) that leads to a constant WVTR value. The WVTR values are presented in Fig. 8. The WVTR of CFs and ENMs were 266.7 and 1332.02 g/m^2^ day, respectively, which are consistent with data have been reported in the literature. The WVTR value of control was 1555.01 g/m^2^ day. Based on these acquired values, ENMs transmitted 85.66% of the water vapor and CFs transmitted 17.15% of water vapor. Indeed, the structural properties of a wound patch-like thickness and porosity as well as hydrophilicity of the biomaterials would determine the WVTR values (Peles and Zilberman 2012).

### Mechanical strength

**Fig. 9:**
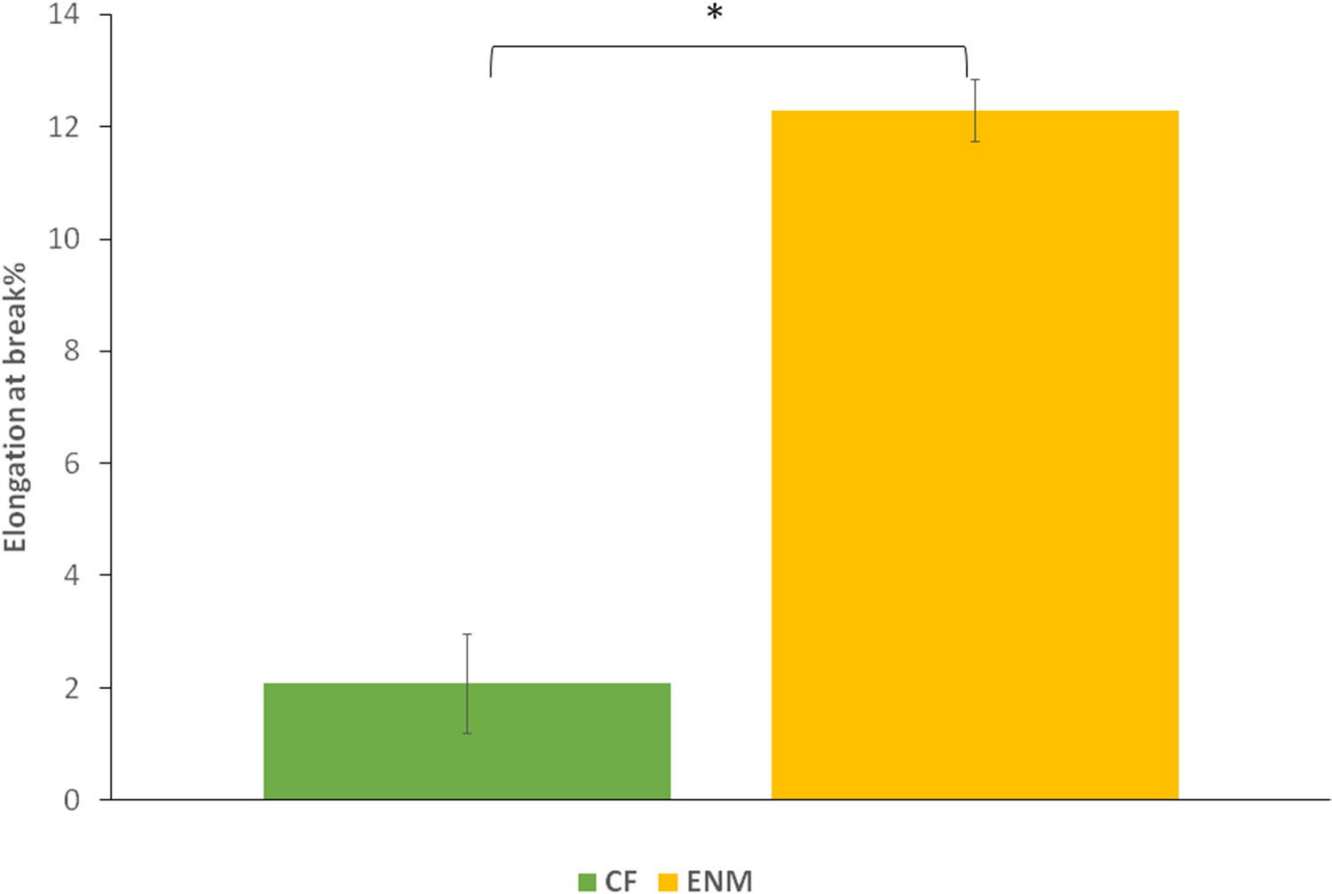
Elongation-at-break percentage of PVA/SPI/glycerol samples (*P *˂ 0.05)

**Fig. 10:**
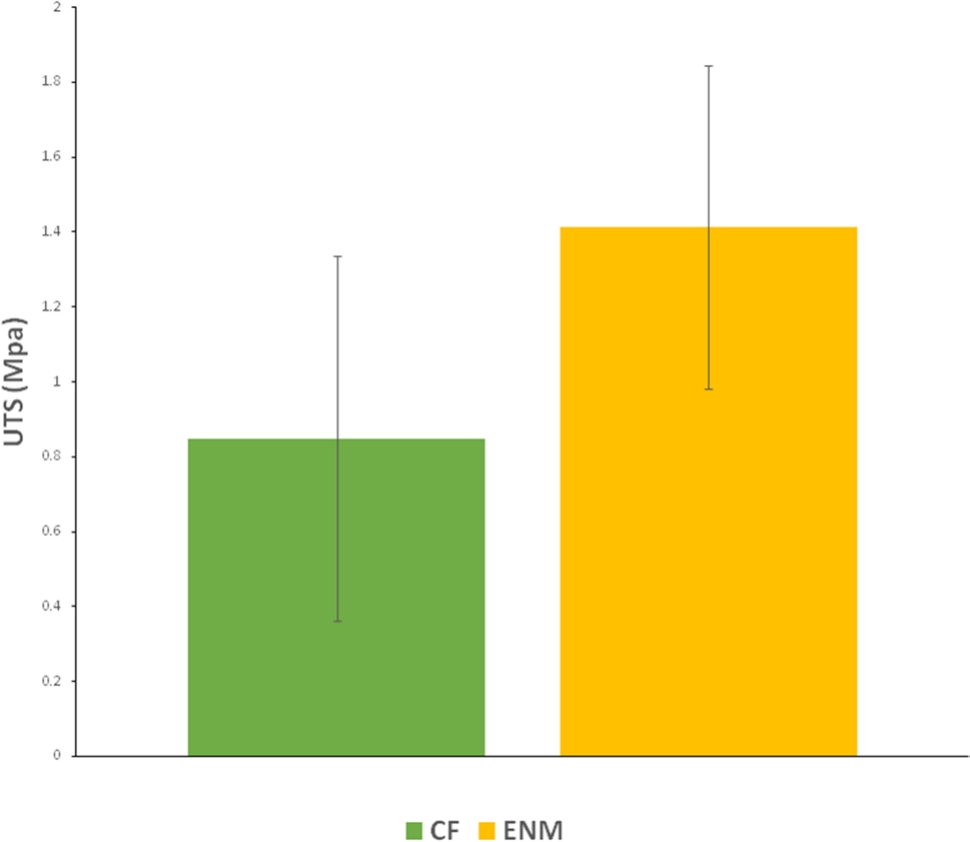
Ultimate tensile strength (MPa) of CF and ENM

The wound protection performance of a wound patch is determined by its mechanical properties. It should withstand stress and not tear apart as well as supporting cell behavior such as cell adhesion, cell spreading and ECM secretion (Peles and Zilberman 2012; Bahrami et al. 2019).The elongation-at-break and ultimate tensile strength (UTS) of CFs and ENMs was assessed using a cross-head tensile tester. Figures 9 and 10 show the percentage of elongation and UTS graphs for both specimens, respectively. *P* value is considered less than 0.05. There is a difference in the percentage of elongation between CFs and ENMs. As the result showed, ENMs presented higher elasticity and UTS value. This result was expected in that with the same composition material, fibrous structures have higher tensile properties (Delyanee et al. 2017).

### Cell response

#### Cell adhesion

**Fig. 11:**
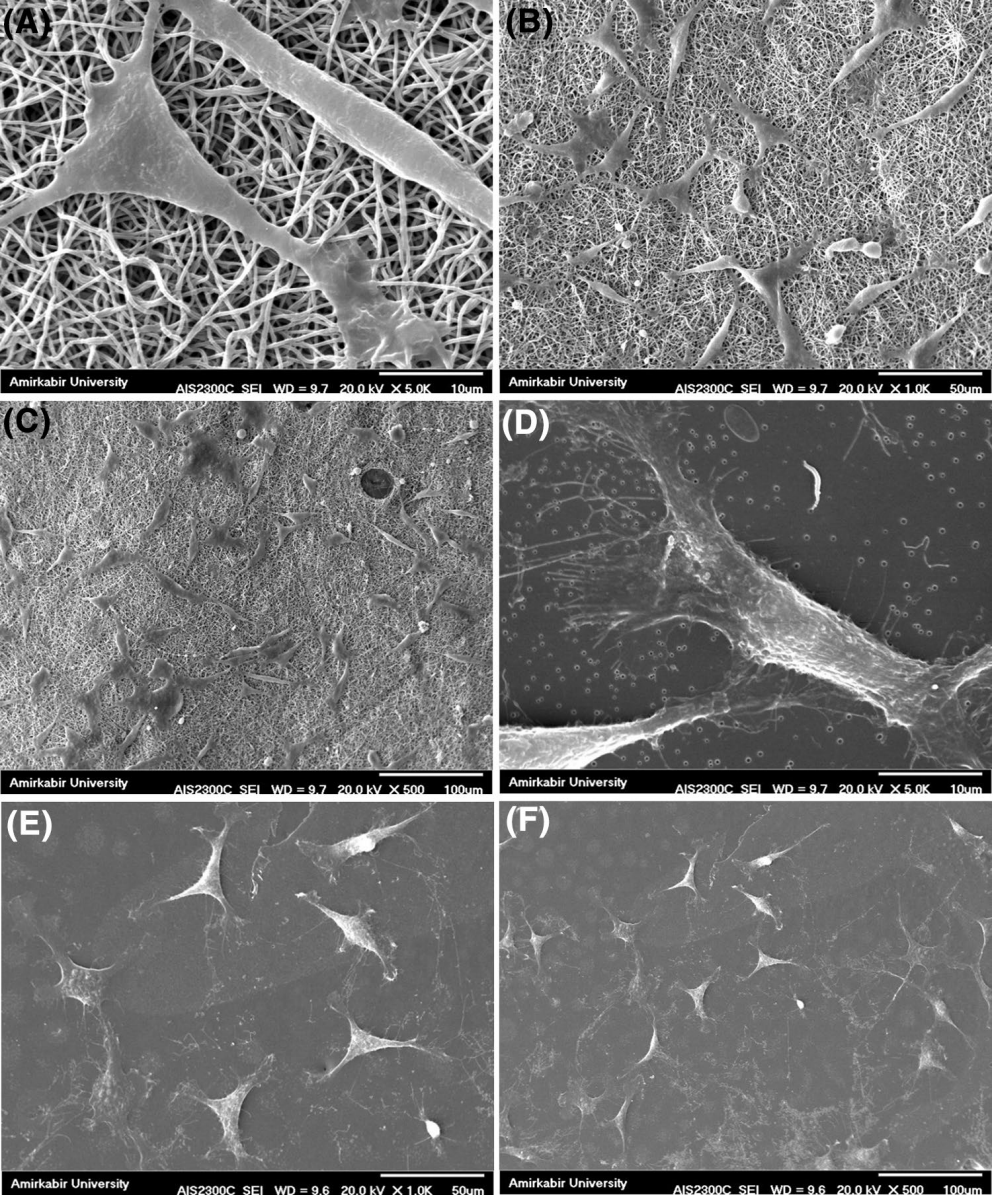
SEM images of cell adhesion on the surface of the ENM (**a**–**c**) and CF (**d**–**f**)

The surface of a biomaterial should be optimal for cell adhesion and cell supporting behavior, and this is perquisite for a successful application (Bahrami et al. 2019). As the components of both CFs and ENMs are biocompatible, the reasonable cell attachment was expected. Figure 11 shows SEM images of cell adhesion on CFs and ENMs after 48 h. They show that both samples have a good and suitable surface for cell adhesion and spreading. In comparison with CFs, ENMs’ structure, as it was expected, showed better cell adhesion and support, but it was necessary to examine and support this assumption quantitatively. As it is shown in the pictures, with the same magnification, there are more cells attached and spread on the surface of ENMs patches. This can be attributed to the structure of ENMs, as their porous structure would provide sufficient nutrition for cells through the pores. Whereas, the cell adhesion occurred only on the edge of the CFs and the center of the samples were cell free, since cells did not have enough access to the nutrition available in the medium. (The SEM images of CFs were taken from the edge of the films).

#### Cell viability

**Fig. 12:**
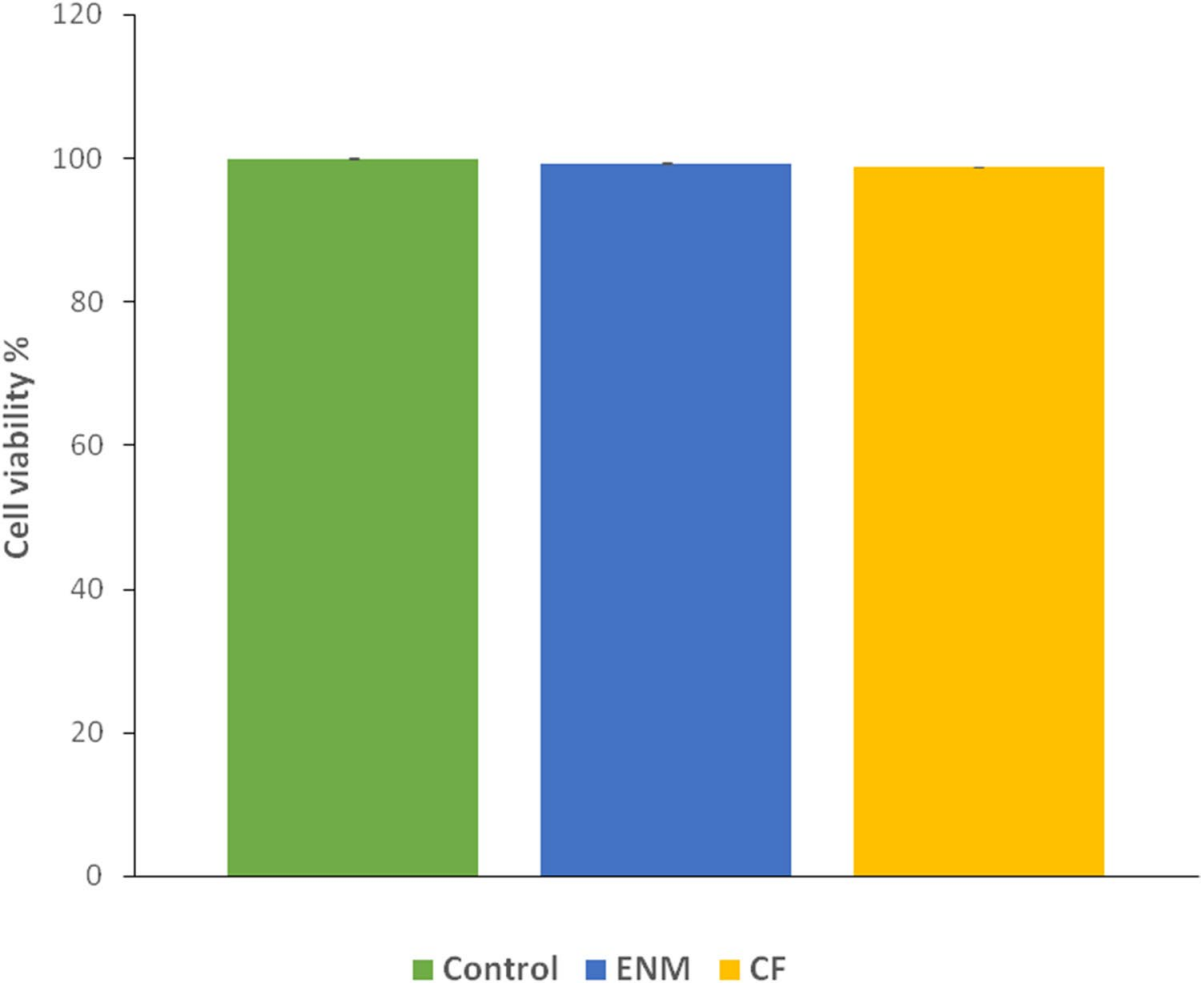
Cell viability percentage of specimens after 72 h

MTT assay is essential, as it provides insight about the compatibility of a material for biomedical application (Bahrami et al. 2019). Both CFs and ENMs extractions after 24 h were used for indirect MTT assay. After 72 h contact between cells and samples, an appropriate medium for cell activity and viability was provided. In fact, no cytotoxic effect was observed and the cell viability percentage was more than 98% (Fig. 12). Due to porosity of the ENMs, the nutrition available in the culture medium were available for cell through the pores and that is why the cell viability of the ENMs were higher than CFs.

## Discussion

The features of the CFs are attributed to the presence of glycerol and PVA in the structure, since the previous literature reported that pure SPI films were not flexible (Su et al. 2007). By comparing to the previous studies, the average fiber diameter decreased as glycerol was added (Cho et al. 2010). This result was expected in that by being positioned within the three-dimensional network of the protein by a plasticizer, the free volume will be increased and the mobility of the polymer chains will be easier (Koshy et al. 2015). Therefore, it would have an effect on the viscosity of the solution and simultaneously would have a direct effect on the fiber diameter.

FTIR spectrum of PVA confirmed the presence of all peaks related to hydroxyl and acetate groups. The spectrum of SPI presents the presence of amino acid proteins. The results of FTIR spectra showed the presence of amino acid proteins and glycerol in the bulk of CFs and ENMs. In addition, the spectrum confirmed the presence of GA in the structure of the specimens, so the crosslinking was successful.

Due to the porous structure of the ENMs, it was expected that their simulated exudate solution uptake percentage would be higher than CFs. The dense structure of the CFs did not allow for the water uptake. The greater amount of ENM’s water uptake makes it a suitable wound dressing for highly draining wounds and bleeding wounds. CFs and ENMs exhibited different stages of water uptake during the 48 h of immersion in the simulated exudate solution. There was an increasing uptake which is due to the influx of water into the specimens. The descending pattern in the water uptake is as the result of a spring-like contraction in the crosslinked network and secretion of glycerol molecules and water. The next escalations in the water uptake pattern after every decrease are because of the polymeric chain relaxation and a swollen matrix (Peles and Zilberman 2012).

The WVTR value of a normal skin is about 204 g m^−2^ day^−1^,and in severe burns, this value may reach 5138 g m^−2^ day^−1^(Peles and Zilberman 2012). The high WVTR value of ENMs is clearly due to the porous nature of the fibers that allows more evaporative water loss through itself. Indeed, the structural properties (thickness and porosity) would determine the diffusion of water through membranes and material’s hydrophilicity of the specimens would affect the WVTR values (Peles and Zilberman 2012). Keeping moisture in wound bed is crucial. CF’s low WVTR value would obstruct the wound healing due to poor drainage of the absorbed exudation.

It was reported that polyol-based plasticizers (compounds containing multiple hydroxyl groups) would reduce the mechanical strength and increase the flexibility when it forms hydrogen bonds and penetrates between protein chains (Peles and Zilberman 2012). In crosslinking using GA, aldehyde groups on both sides react with amino groups resulting in forming a network, and by this mean, it will increase the mechanical strength. The presence of glycerol may lead to an unwanted interaction between GA and glycerol itself; therefore, it may block the interaction between active aldehyde groups and amino groups of the protein resulting in a brittle behavior. Besides this, alcoholic nature of PVA and the presence of hydroxyl groups can also react with glycerol and may generate water as its side products; therefore, it may lead to a reduction in the activity of the cross-linker agent.

Both CF and ENM supported the cytocompatibility. It is known that topography of the surface plays an important role in the cell’s behavior. Nanostructure of the fibers would result in an increase in the surface area which would ease the accessibility of cells to absorb the serum protein (Fukushima 1969).
